# The profiles of miR-4510 expression level in breast cancer

**DOI:** 10.1038/s41598-022-25292-1

**Published:** 2023-02-08

**Authors:** Sevan Omer Majed, Suhad Asad Mustafa

**Affiliations:** 1 Biology Department, College of Education, Salahaddin University-Erbil, Erbil, Iraq; 2 General Directorate of Scientific Research Center, Salahaddin University-Erbil, Erbil, Iraq

**Keywords:** Cancer, Computational biology and bioinformatics, Molecular biology

## Abstract

MicroRNA that is abnormally produced in breast cells can disrupt biological processes, which can lead to cancer. This study aims to screen differentially expressed genes (DEGs) and ncRNAs (DEncRNAs) in the formalin-fixed paraffin-embedded (FFPE) tissues of breast cancer (BC) as compared with the normal adjacent tissues (NAT), and identify miR-4510 as a novel biomarker of BC. This study looked at differentially expressed genes (DEGs) using MACE-Seq and differentially expressed ncRNAs (DEncRNAs) using the small RNA-Seq. Real-time qPCR was used to determine the level of expression of miR-4510. In this study, MACE-Seq results showed that 26,795 genes, with a *p*-value < 0.05, were differentially expressed in BC paraffin tissues as compared with NAT. Small RNA-Seq results revealed that 1326 ncRNAs, with a *p*-value < 0.05, were differentially expressed. We confirmed that miR-4510 was significantly down-expressed (*p*-value = 0.001) by qRT-PCR in the paraffin tissue of 120 BC patients. Based on eleven computational prediction programs, *TP53*, *TP53INP1*, *MMP11*, and *COL1A1* for the miR-4510 were identified as miR-4510 targets. The MACE-seq result showed that the gene of TP53 (*p*-value = 0.001) and TP53INP1 (*p*-value = 0.02) was significantly down-regulated, but the gene of *MMP11* (*p*-value = 0.004) and *COL1A1* (*p*-value = 0.0001) was significantly over-expressed in 20 paired specimens of the BC and NAT. We discovered that a single SNP inside the miR-4510 binding site occurred only in BC, in which Guanine (G) changed into Adenine (A). Two SNPs outside the miR-4510 binding site occurred, and Guanine (G) in both BC and NAT was changed into Thymine (T), as compared to the reference sequence (RefSeq). Overall, our results suggested that miR-4510 functions as a tumor suppressor in the BC. Mir-4510 may act as a tumor suppressor, however additional experimental data is needed to corroborate these assumptions and can be exploited as a biomarker for BC.

## Introduction

Breast cancer (BC) is the most complex cancer observed in women and constitutes about 33.8% of newly diagnosed patients by 2040^1,2^. Although the survival rate of BC in the eastern world is decreasing, those in western countries are still increasing^1,3^. This type of cancer is the second most dangerous cancer-associated mortality after skin cancer, particularly in the US and middle east nations^4–6^. Genomic and epigenetic aberrations are found to be the main cause of the development of breast carcinogenesis. A new study has found that several etiological reasons are responsible for this situation. About 10% of BC are genetic; while, 90% are environmental. Several candidate genes are implicated in the BC. Especially, *BRCA1* and *BRCA2* mutation carriers have a 45–80% lifetime risk of BC. and are the two common genes associated with 90% of subtypes of inherited BC. Other candidates, such as *PTEN*, *P53*, *PALB2*, *CHEK2*, *ATM*, *NF1*, and *STK11,* have also played an important role in the hereditary BC subtypes. The main cellular mechanisms that participated in cell growth and proliferation are changed by dysregulation of gene expression^7^. Therefore, examination and analysis of the genes are helpful to identify mutations in the genes or genome and recognize novel alterations or alleles related to the BC. Some molecular machines, such as quantitative Real Time-Polymerase Chain Reaction (qRT-PCR), DNA sequencing, MACE-sequencing, DNA microarray, and next-generation DNA sequencing, are the most common methods performed to screen variations of the BC genes. These tests will be required to exploit prognostic, and diagnostic cancer biomarkers^8,9^.

Recently, studies have shown that gene expression tests in BC can be used in medicinal choice for the treatment of BC subtypes. Using molecular techniques, the genomic screens have contributed to the more detailed methods of categorizing the BC, depending on the expression of their genes, by dividing them into 4 major molecular subtypes^10–13^. The molecular subtypes are luminal-A and -B (including Human Epidermal Growth Factor Receptor 2 (HER2 +/−)), (HER2 +), and triple-negative (TN). Overall, the diagnosis of these molecular subtypes is crucial for treatment decision^10,14^.

MicroRNAs (miRs or miRNAs) are short (about 19-20nt) non-coding single stranded RNAs^15,16^, which have been found to have an effect on the cancer development caused by the differential expression of cancer-causing genes , Fromm et al., reported^17^. The miRNAs play a vital role in both the post-transcriptional gene expression and the silencing of mRNA molecule, by binding to the 3′-UTR of target mRNAs. Thus, they can either cleave mRNA into 2 pieces or destabilize mRNA by removing its poly (A) tail^15,18^. The complementary base pairing between the miR and its mRNA is important for regulating the mRNA level and hence protein expression. Any mutation in the binding site of either the miRNA or its mRNA may decontrol gene expression and hence cause the development of different pathological events, such as carcinogenesis^19^.

Studies have recently found that miRs are numerous in human cell types and implicated in different pathological and biological mechanisms; such as cell growth, differentiation, proliferation, migration, invasion, and death^16,20,21^. Therefore, once one or more miRs was low or high expressed, cancer can be developed. In human malignant tissues, numerous miR genes were observed to function as oncogenes or tumor suppressors. OncomiRs are implicated in stimulating genes that control the cell development and apoptosis process. Once one of the oncomiRs is overexpressed, the growth of tumor happened. Tumor suppressive miRs (TS-miRs) are responsible for silencing genes that regulate cell development and apoptosis^22,23^. Like oncomiRs, once one of the TS-miRs is down-expressed in normal cells, the cells undergo abnormal growth^13,24^. Several researches revealed that the majority of miRs are directly involved in modulating cellular processes; cell development, proliferation, migration and apoptosis^13,16,17^. As a result, several human cancer types; such as breast, lung, colon, skin, leukemia, liver, colorectal cancer, and brain, are developed^20,23^. For instance, many miRs in human BC have been identified to exert key functions in cellular behaviors and cancer formation^25^. Two studies revealed that miR-181a, miR-26b, miR-23a, miR-145, miR-125b, miR-155, and miR-21 in BC cells were significantly low expressed in BC cells. A correlation was detected between these miRs and pathologic characters; estrogen and progesterone receptor expression levels, cell development and cell proliferation^26,27^. Furthermore, Lu et al. demonstrated low levels of miR-140-5p in BC cell lines and clinical tissues. Up-regulation of miR-140-5p expression using miRNA mimic suppressed the BC aggressiveness and also angiogenesis in vitro and in vivo. Suppression of VEGFA expression was targeted by miR-140-5p. Thus expression of CD31, Ki-67, and MMP-9 was reduced through inhibition of the VEGFA expression^28^. Although, there is insufficient evidence on the level of miR-4510 expression and its targets in BC, the profile of the expression has been studied in human malignancies. For example, miR-4510 in colorectal cancer was found to be down-expressed^29^; whereas, miR-4510 in stage IA lung adenocarcinoma was identified to be overexpressed^30^. target Notch3 mRNA in ovarian cancer^31^.

In this study, total RNA elements in formalin-fixed paraffin-Embedded (FFPE) tissues of BC are amid to be firstly analyzed to detect the differential expression profiles of transcript mRNAs and small non-coding RNAs (sncRNAs) using Massive Analysis of cDNA Ends (MACE) and TruQuant techniques, respectively. Then, the differential expression of miR-4510 will be tested using quantitative Real-Time Polymerase Chain Reaction (qRT-PCR). Target predicted genes that regulated by miR-4510 will be searched in computational prediction programs. The miRNA-4510 mature sequence will be sequenced to identify the single nucleotide polymorphism (SNP). Differential expression level of these targeted genes will be analyzed in MACE results. To investigate the biological function of miR-4510 in BC, gene enrichment analysis can be performed for the predicted target genes of miR-4510 to initially determine its role, such as David analysis. Overall, our current study demonstrated that miR-4510 may be a novel biomarker for BC detection.

## Material and method

### Collection of FFPE-blocks of BC samples

One hundred and twenty paired samples were experimented in this study. Samples were taken from cases who were not exposed to radiotherapy, chemotherapy and hormonal therapy. we collected 120 paired samples of formalin fixed paraffin embedded (FFPE) tissues from Kurdish cases with BC at laboratories of clinical pathology; such as Luay and Al-Mufti. For each patient, two paraffin blocks were taken (one malignant tissue and one normal adjacent tissue (NAT) as a control). Normal tissues were taken 2 cm away from the malignant region. The malignant and non-malignant areas were determined using Eosin and Hematoxylin stains. Clinical properties of the cases were gained. Table 1 provided the detailed information on the property of participants.

**Table 1:** Features of clinical pathology of 120 BC participants.

Pathological feature	Exploratory No. of cases (%)	Confirmation No. of cases (%)
**Age**		
< 50	11 (55)	75 (62.5)
≥ 50	9 (5)	36 (37.5)
**BMI**		
≤ 20	1 (5)	19 (15.84)
20–25	9 (45)	40 (33.33)
≥ 25	10 (50)	61 (50.83)
**Tumor size (**cm**)**		
≤ 2	0	37 (30.83)
2–5	5 (25)	68 (56.66)
≥ 5	15 (75)	15 (12.51)
**Tumor grade and stages**		
IB	0	10 (8.3)
IIA	0	25 (21)
IIB	0	50 (41.7)
IIIA	5 (25)	15 (12.5)
IIIB	7 (35)	18 (15)
IIIC	8 (40)	2 (1.5)
**Lymph node metastasis**		
Yes	20 (100)	88 (73.33)
No	0	32 (26.67)
**Lymphatic invasion**		
Yes	20 (100)	85 (70.83)
No	0	35 (29.17)
**Venous invasion**		
Yes	20 (100)	77 (64.17)
No	0	43 (35.83)
**Biomarkers**		
TN	11 (55)	67 (50.83)
ER + and PR +	9 (45)	28 (23.33)
ER − and PR −	0	15 (12.5)
ER + and HER2 +	0	10 (8.33)
**Ki67 status**		
High	15 (75)	80 (66.7)
Low	3 (15)	28 (23.3)
Not available	2 (10)	12 (10)
**Technique**		
MACE-seq and small RNA-seq	20 (100)	
qPCR		120 (100)

*BMI* Body mass index, *TN* Triple negative, *ER* Estrogen Receptor, *PR* Progesterone Receptor, *HER2* Human Epidermal Growth Factor Receptor 2.

### Isolation of total RNA elements for transcript mRNAs and sncRNAs

Total RNA sequencing was performed to analyze differentially expressed genes (DEGs) and differentially expressed non-coding RNAs (DEncRNAs). The procedure of total RNA isolation and ncRNA separation was performed for the paraffin tissues of 20 cases from GenXPro GmbH in Germany. First, cancerous and non-cancerous tissues in histopathological laboratory were firstly determined using Eosin and Hematoxylin stains. Ten micrometers for each sample were then deparaffinized using Xylene twice for 5 min each and different grades of ethanol (100%, 90% and 70% ethanol) for 2 min each. Next, total RNA elements were extracted as described in the protocol of MACE-seq kit (GenXPro GmbH, Frankfurt, Germany). After total RNA extraction, the small RNA molecules were separated using TrueQuant small RNA kit. Analyses of MACE-sequencing for transcript mRNAs and small RNA sequencing for the ncRNAs were then carried out for 20 samples (Table 1).

### Generation of libraries for MACE- and small RNA sequencing

After PCR performance, the sequences of mRNA transcript for the building of the MACE-libraries and the small RNA molecule for the building of sncRNA libraries were used. Libraries were built for 20 paired samples (20 BCs and 20 NATs). MACE-Kit was used to purify the mRNA transcripts from the total RNA (GenXPro GmbH, Frankfurt, Germany). cDNA synthesis was then performed using the SuperScript® III First-Strand Synthesis System. Next, the cDNA products were subjected to streptavidin beads. Then, the products were randomly fragmented to be about 300 bps. After elimination of unbound fragments, poly-A tails and beads, the purified transcripts were sequenced. MACE-tag population was then performed. Each transcript is represented by only one TrueQuant barcode (tag). Each read detects one transcript. The copies of a transcript were then eliminated. The barcoded reads were simultaneously sequenced in a lane of an Illumina Hiseq2000 with 1 × 75 bps.

For the preparation of ncRNA libraries, flashPAGE™ Fractionator System (Life Technologies) was used to purify them. The ncRNA molecules were directly attached by TrueQuant tags, basically as mentioned by Hafner and his colleagues^32^. Then, we used Hiseq2000 technique to utilize sequence the small molecules adding p5 and p7 adapters. Next, these adapters were eliminated from the reads before the organization of reads. For the alignment of reads, bowtie2 alignment tool was later applied, which aligned the arranged reads to the reference sequences. For the normalization of the aligned reads, we normalized them to measure different sequencing depths. The Limma package in R language was utilized to searching differentially expressed ncRNAs (DEncRNAs) between BC and NAT. We used Bonferroni in the multtest package to adjust the *P*. value into the FDR. We also used the FDR < 0.05 and |log2 FC|> 0.5 as the cutoff criteria for the DEncRNAs. The information on the accession number, *P*. value, location, Log2 fold change (Log2FC), and False Discovering Rate (FDR) of these 29 miRNAs was revealed in Table 2. Moreover, the expression level was found to be significantly lower in cancerous tissues than non-cancerous tissues. Thus, miR-4510 was focused to confirm expression level using qRT-PCR technique in this study.

**Table 2:** Marked down-regulated microRNAs in BC compared with NAT.

miR	miRBase accession	Location	Log2FC	*P*. value
**miR-4510**	***MIMAT0019047***	**15q14**	** − 3.1805**	**0.002**
miR-100-5p	*MIMAT0004512*	11q24.1	− 1.2349	0.0282
miR-30b-5p	MIMAT0000420	8q24.22	− 1.3254	0.0245
miR-30d-5p	*MIMAT0000245*	8q24.22	− 0.9730	0.0389
miR-410-5p	*MIMAT0026558*	14q32.31	− 0.6780	0.0489
miR-125a-5p	*MIMAT0000443*	19q13.41	− 1.4153	0.0213
miR-125b-5p	*MIMAT0000423*	11q24.1	− 2.0041	0.0372
miR-130a-3p	*MIMAT0004593*	11q12.1	− 2.0168	0.0104
miR-133a-5p	*MIMAT0026478*	18q11.2	− 2.3885	0.022
miR-143-5p	*MIMAT0004599*	5q32	− 1.2823	0.0308
miR-204-5p	*MIMAT0000265*	9q21.12	− 4.0627	0.086
miR-25-5p	*MIMAT0004498*	7q22.1	− 1.2630	0.0393
miR-214-3p	*MIMAT0000271*	1q24.3	− 0.8746	0.044
miR-21-3p	*MIMAT0004494*	17q23.1	− 0.0365	0.0295
miR-30a-3p	*MIMAT0000088*	6q13	− 0.2358	0.0838
miR-934	*MIMAT0004977*	Xq26.3	− 3.0931	0.0417
miR-99b-5p	*MIMAT0000689*	19q13.41	− 0.1667	0.05
miR-423-5p	*MIMAT0004748*	17q11.2	− 0.5727	0.0514
miR-10b-5p	*MIMAT0000254*	2q31.1	− 1.2852	0.0261
miR-374b-5p	*MIMAT0004955*	Xq13.2	− 0.2420	0.0435
miR-505-5p	*MIMAT0004776*	Xq27.1	− 1.5956	0.011
miR-532-3p	*MIMAT0004780*	Xp11.23	− 0.7496	0.0412
miR-451a	*MIMAT0001631*	17q11.2	− 0.41900	0.0311
miR-455-5p	*MIMAT0003150*	9q32	− 2.5081	0.0305
miR-664b-5p	*MIMAT0022271*	Xq28	− 0.5081	0.0289
miR-92a-1-5p	*MIMAT0004507*	13q31.3	− 0.2085	0.0472
miR-624-5p	*MIMAT0003293*	14q12	− 0.0931	0.0498
miR-664a-3p	*MIMAT0005949*	1q41	− 0.9249	0.0104

Significant values are in bold.

### Deparaffinization of paraffin tissue

In the present study, miR-4510 was selected from the ncRNA results for the determination of differential expression using the quantitative Real Time-polymerase chain reaction (qRT-PCR) technique. A differential expression analysis of miR-4510 was performed on 100 paired samples of paraffin tissues (100 NAT and 100 BC paraffin tissues). Cancerous and non-cancerous tissues from these paraffin blocks were histologically determined using Eosin and Hematoxylin stains. This experiment was conducted at Salahuddin University Research Center (SURC). 20 mg of the paraffin tissue was measured and put in the microcentrifuge tube for each experiment. Prior to the total RAN preparation, the paraffin tissue was deparaffinized by incubating that for 5 min in Xylene. Then the tissue was heated at 50 °C for 10 min. The tissue was then centrifugated for 2 min at 14,000 rpm. With absolute ethanol, 90% and 70% ethanol for 2 min each, the pellet was rehydrated. The rehydration and centrifugation have again been repeated. The tissue pellet was finally air-dried at ambient temperature for 20 min. Using a PowerGen 50 for 30 s each, the deparaffinized pellet was then sonicated.

### Preparation of total RNA and cDNA for miR-4510 expression determination

The protocol for total RNA preparation was performed as mentioned in NORGEN BIOTEK CORP. Using FFPE RNA/DNA Purification Plus kit (Cat. No. 54300, NORGEN BIOTEK CORP, Canada), total RNA molecules were extracted. Then Nanodrop machine was used to know and evaluate the total RNA concentration. All the samples used had a great concentration of RNA. Complementary DNA (cDNA) for them was generated using the miRNA All-In-One cDNA Synthesis Kit (Cat. No. G898, abmgood company, US). In this experiment, 5 μl of DNA template was mixed with 15 μl of all reagents, as described in the company protocol.

### Differential expression identification of miR-4510 using qRT-PCR

In this experiment, all products of the RT-qPCR were bought from abmgood company, US. For each well, the 20 μl of total reagents was added. The reagent included 1.5 μl of template cDNA, 0.5 μl for each of reverse and forward primers (Cat.No.: MPH02746), 10 μl of BrightGreen (Cat. No. MasterMix-mR), and 7.5 μl of nuclease-free water. For determination of miR-4510 differential expression, 2 universal miRNA primers were utilized, which were the SNORD44 primers (Cat. No. MPH0003), and U6-2 primers (Cat. No. MPH0001). The qRT-PCR reaction was set up for the following 3 steps. Enzyme activation was first set up for ten mins at 95 °C. Then 40 cycles were set up for DNA template separation for 10 s at 95 °C, annealing for 20 s at 63 °C and elongation for 20 s at 72 °C.

### Selection of putative targets regulated by miR-4510

Eleven computational predictions of MicroRNA Target Genes have been checked for the most common miR-4510 putative targets (Table 2). Four predicted genes (*TP53*, *TP53INP1*, *MMP11*, and *COL1A1*) were determined to possess binding sequence to miR-4510 seed site*.* These four genes were the intersection of all database results for target genes. Expression level of these genes were identified in the MACE-Seq results. GraphPad Prism, Version 8.0.1 was applied to show the differential expression of them.

### GO gene enrichment analysis for target genes of miR-4510

To investigate the biological function of miR-4510 target genes in the BC cells, Gene Ontology (GO) enrichment analysis was performed using DAVID analysis (https://david.ncifcrf.gov/summary.jsp).

### Survival analysis

For displaying the role of differential expression of miR-4510 target genes on participant survival, expression data of only those targeted genes and miR-4510 was downloaded from TCGA using Bioconductor tool. R tool was then used for generating the Kaplan–Meier (KM) survival plots. Based on the median expression of mRNA levels and log-rank test, the BC patients were divided into two groups; high expression class, and low expression class. The data was downloaded on 3 August 2021. For future studies, the data analysis and R scripts will be saved.

### Ethics approval and consent to participate

The Human Research Ethics Committee (HREC) at the Science College at Salahuddin University-Erbil (Approval No.4c/132) followed and approved this study. Informed consent was received in accordance with the requirements of the HREC from all participants. The local ethics committee approved the collection of archived FFPE-blocks from BC and NAT. Participants approved by their local ethics committee received the models from Erbil City.

## Result

### Comparison of differentially expressed genes between BC and NAT tissues

**Figure 1:**
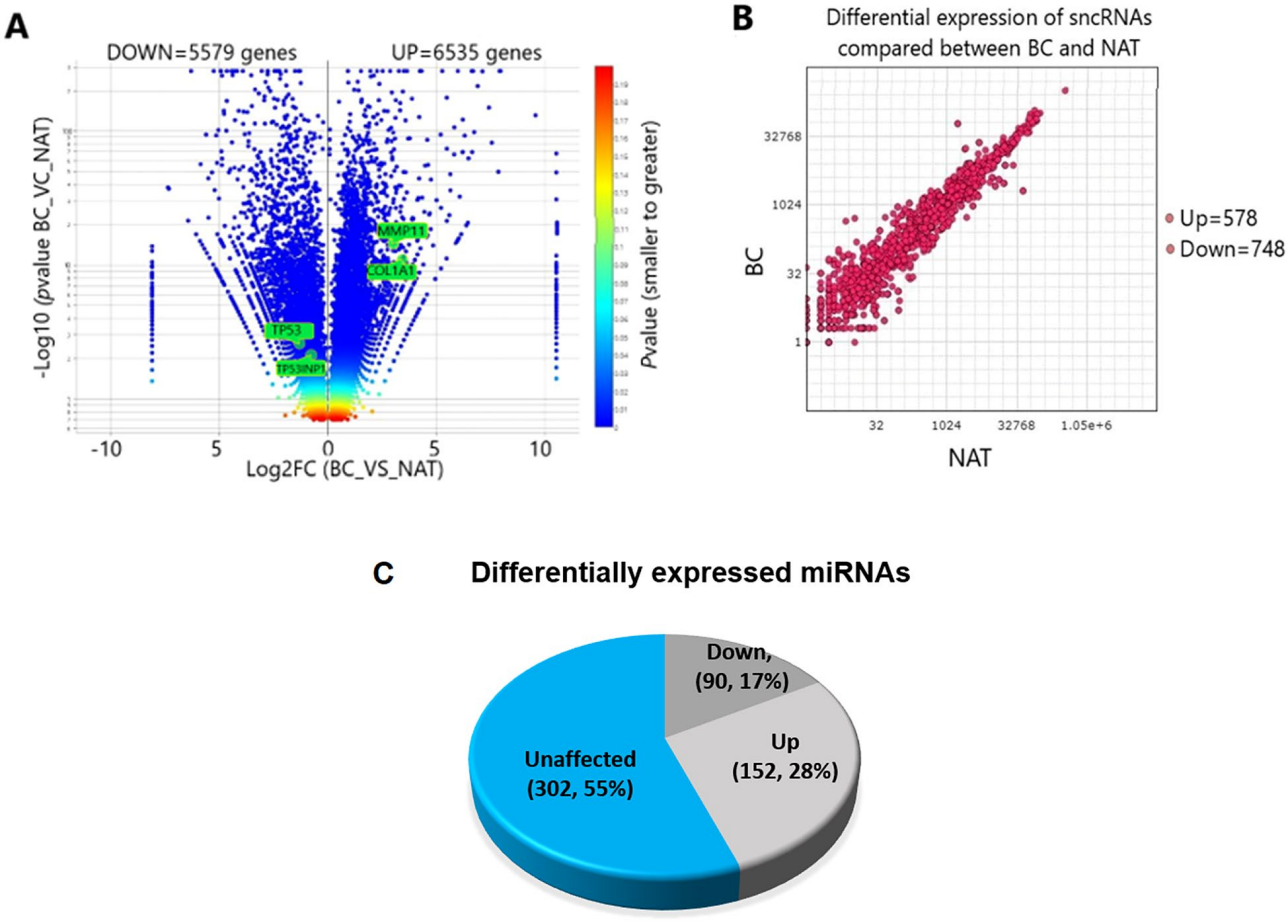
Analysis of the differential expression of mRNAs and small RNAs. (**A**) Volcano plot showed the comparison of the differential expression of 12,114 genes between BC and NAT. each plot denotes only one mRNA transcript. The selected target genes for miR-4510 were pointed and named. (**B**) Scatter plot displayed small RNA differential expression. Each red dot on the plot denoted only one small RNA. (**C**) The total number of miRNA differential expression was compared.

MACE-sequencing technique was used to constructed differential expression profiles. The MACE-seq findings revealed that 26,795 protein-coding genes in BC tissue were differentially expressed compared to NAT tissue, but 12,114 protein-coding genes were displayed on a volcano plot (Fig. 1A). After adjustment to *p*-value < 0.05, SAM software were used to filter out these genes. The volcano plot showed that 6535 genes were up- regulated and 5579 genes were down-regulated. The log2 transformation was used to normalize the differential expression value of these. The *p*-value was considered from smaller (Blue) to greater (Red). Each dot represents only one transcript of observations from the expressed genes. The x-axis represents the normalized values of the expressed gene in the NAT and the y-axis represents the normalized values in the BC. As shown in Table 3, twenty genes were significantly over-expressed in the BC compared to the NAT among the over-expressed genes, and eight genes were significantly down-expressed, as shown in Table 3.

**Table 3:** Comparison of the gene over-expression in BC with NAT.

Genes	Gene ID	Location	Log2FC	*P*. value
**Up-regulated**				
*CPB1*	ENSG00000153002	3q24	7.96048	0.001
*KCNJ3*	ENSG00000162989	2q24.1	7.12804	0.049
*MMP11*	ENSG00000099953	22q11.23	6.69183	0.004
*MYO3B*	ENSG00000071909	2q31.1	6.2235	0.01
*ECM1*	ENSG00000143369	1q21.2	5.27538	0.0012
*PCDH10*	ENSG00000138650	4q28.3	5.20281	0.03
***COL1A1***	**ENSG00000108821**	**17q21.33**	**5.12521**	**0.0001**
*ADGRB2*	ENSG00000121753	1p35.2	4.7562	0.015
*IFI6*	ENSG00000126709	1p35.3	2.95573	0.0193
***MMP11***	**ENSG00000099953**	**22q11.23**	**4.21504**	**0.04**
*ABCC5*	ENSG00000114770	3q27.1	3.77894	0.05
*HSH2D*	ENSG00000196684	19p13.11	3.70495	0.0431
*SERHL2*	ENSG00000183569	22q13.2	3.65214	0.0012
*TYMP*	ENSG00000025708	22q13.33	2.87753	0.0018
*ATP2A3*	ENSG00000074370	17p13.2	3.45979	0.0491
*EVL*	ENSG00000196405	14q32.2	3.27742	0.0271
*MISP*	ENSG00000099812	19p13.3	3.14003	0.0328
*SERHL*	ENSG00000172250	22q13.2	3.1396	0.0081
*SCUBE2*	ENSG00000175356	11p15.4	2.75852	0.0469
*MLPH*	ENSG00000115648	2q37.3	− 2.35618	0.017
**Down-regulated**				
***TP53***	**ENSG00000141510**	**17p13.1**	** − 4.31853**	**0.001**
***TP53INP1***	**ENSG00000164938**	**8q22.1**	** − 3.58318**	**0.02**
*MAST4*	ENSG00000069020	5q12.3	− 2.38064	0.0122
*SMIM22*	ENSG00000267795	16p13.3	− 2.29172	0.0134
*CXCL14*	ENSG00000145824	5q31.1	− 2.27225	0.002
*DOPEY2*	ENSG00000142197	21q22.12	− 2.2232	0.0259
*DUSP4*	ENSG00000120875	8p12	− 2.13023	0.0482
*KRT19*	ENSG00000171345	17q21.2	− 0.948134	0.045

Significant values are in bold.

### Putative target genes of miRNA-4510

Based on the eleven computational prediction sites, the gene of *MMP11*, *COL1A1, TP53* and *TP53INP1* was validated and found to have a complementary base pairing in the 3′-UTR regions with miR-4510 seed site. These sites are established to predicted either microRNAs to target genes or target genes to microRNAs. Among the results of MACE-Seq, twenty-eight genes were significantly differential expressed in the BC compared with NAT. The gene ID, genomic location, Log2FC and *p*-value about the 28 up- and down-regulated genes were exhibited in the Table 3. With help of these sites, four of the 28 genes were found to be the target of miR-4510. Table 4 showed eleven computational programs of putative miRNA targets that were applied to find out the miR-4510 targets. Genes of *TP53*, *TP53INP1*, *MMP11*, and *COL1A1* were detected to be the intersection of all database results for target genes and also had the binding site to the miR-4510 seed sequence. These genes were also pointed out and named on the volcano plot (Fig. 1A).

**Table 4:** Target predicted tools searched for identifying target predicted genes having complementary bases to miR-4510 seed site.

Target predicted sites	Species	Tool properties	Websites
miRTarBase	Human, Mouse, Rat	Conservation, seed location	http://mirtarbase.mbc.nctu.edu.tw/php/index.php
Target scan	Human, Mouse, Fly, Fish, and Worm	Conservation, seed location	http://www.targetscan.org/
TargetMiner	Human, Mouse, Rat, Fly	Conservation, seed location	https://www.isical.ac.in/~bioinfo_miu/TargetMiner.html
MirTar2	Human, Mouse, rat, Dog and Chicken	Conservation, seed location	http://www.mirdb.org/
DIANA	Any	Conservation, seed match, and free energy	http://www.microrna.gr/microT-CDS
miRWalk	Human, Mouse, and Rat	Conservation, seed match and free energy	http://mirwalk.uni-hd.de/
miRmap	Human, Chimpanzee, Mouse, Rat, Cow, Chicken, Zebrafish, and Opossum	Conservation, seed match, and free energy	https://mirmap.ezlab.org/
RNA22	Human, Fruit Fly, Mouse, and Worm	Seed match and free energy	https://cm.jefferson.edu/rna22/
PicTar—Tools4miRs	Human, Mouse, Rat, Fly	Conservation, seed location	https://tools4mirs.org/software/target_prediction/pictar/
mirPath	Human, Mouse, *D. melanogaster*, *C. elegans*, *R. norvegicus*, *D. rerio* and *G. gallus*	Conservation, seed match and free energy	http://snf-515788.vm.okeanos.grnet.gr/index.php?r=mirpath/geneList
Microrna. org	Human, mouse, Fruit Fly, and rat	Conservation, seed match, free energy	http://www.microrna.org/

### Building of differential expression of small RNAs via small RNA-Seq technique

Analysis of differentially expressed non-coding RNAs (DEncRNAs) in the cancerous tissues was conducted to identify either up-regulated or down-regulated compared to the non-cancerous tissues. Here, SAM software was used to filter out 1326 non-coding RNA molecules, including miRNAs, lncRNAs, piRNAs, rRNAs, tRNAs, and circRNAs. Then, standardization for the raw data was executed by applying log2 fold-changes as shown on a scatter graph (Fig. 1B). 748 ncRNA molecules were down-expressed, but 578 ncRNAs were overexpressed among 1326 DEncRNA molecules. Each red dot denotes only one ncRNA molecule. The y-axis represents the BC data, and the x-axis represents the NAT data. We found that 544 microRNAs were differentially expressed among these DEncRNA molecules. Overexpressed miRNAs were 152 (28%) and down-expressed miRNAs were 90 (17%), but unaffected miRNAs were 302 (55%) in the BC cells as compared with NAT among 544 miRNAs (Fig. 1C). Among the 90 down-expressed miRNAs, 27 miRNAs were significantly down-regulated in BC as compared to NAT. In this study, miR-4510 was focused to confirm expression level using RT-qPCR technique and sequence alterations in the BC compared against the NAT, as shown in light gray color in Table 2. The instruction on the ID number, *p*-value, genome loci, Log2FC, and FDR of these 27 microRNAs was shown in Table 2.

### miR-4510 differential expression confirmation

**Figure 2:**
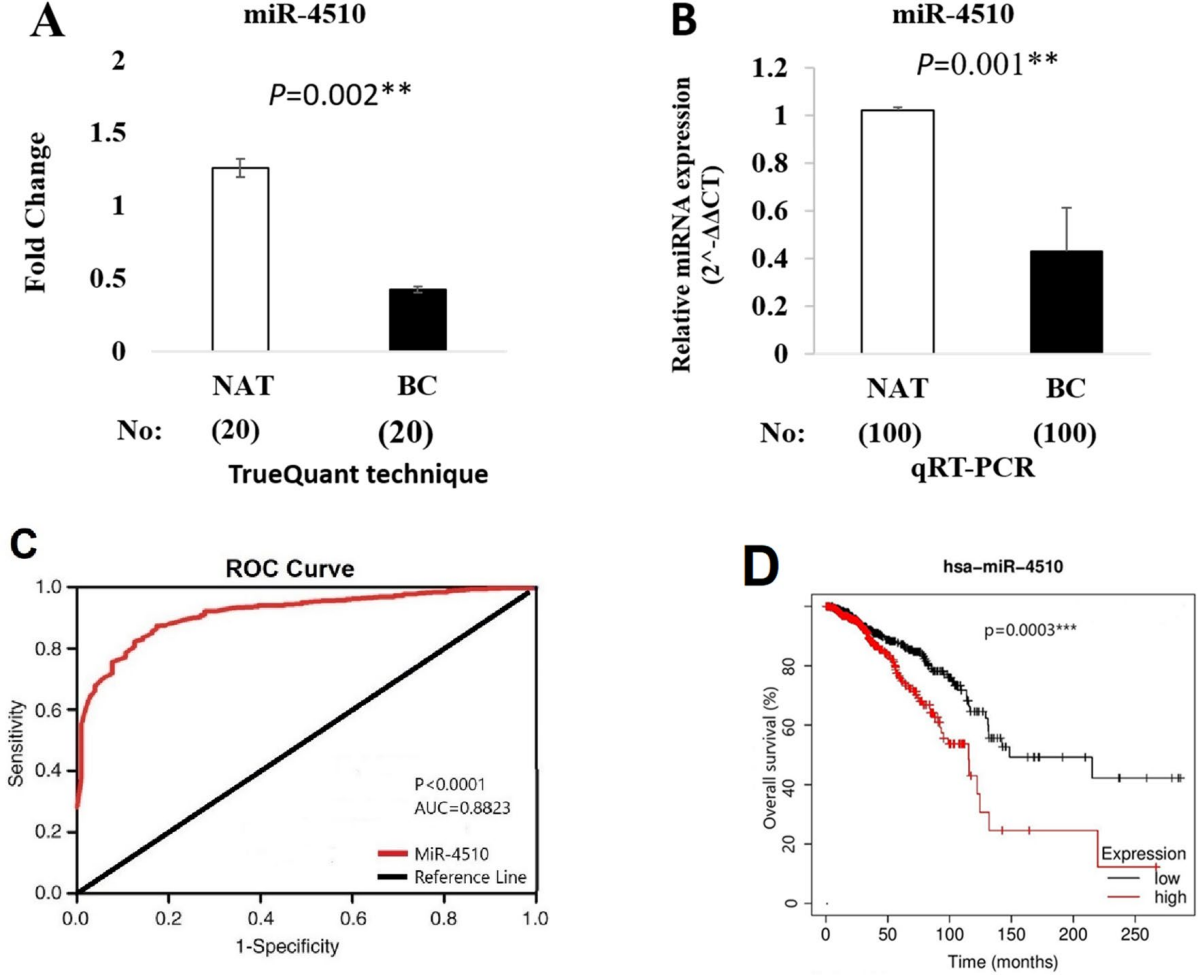
(**A**) Differential expression of miR-4510 in BC was compared with NAT using the MACE-seq method. (**B**) Differential expression of miR-4510 in BC was compared with NAT using the RT-qPCR method. (**C**) ROC curves for miR-4510 based on the RT-qPCR data derived from the breast cancer and normal tissues. The diagram designed the sensitivity (true positive rate) against 1—specificity (false-positive rate) over all possible ΔCT values. The AUC number show that the two classes could be differentiated by expression analysis of the biomarker. (**D**) K-M analysis of an association between miR-4510 expression and patient overall survival.

Based on the outcomes of ncRNA molecules, the expression level of miR-4510 in 20 paired paraffin samples of patients with BC was significantly lower than in NAT, *p*-value = 0.002 (Fig. 2A). For the confirmation of this result, differential expression analysis was confirmed using an RT-qPCR machine in 100 paired paraffin samples of other patients. The RT-qPCR results confirmed that the expression level of miR-4510 in the BC tissue was markedly lower than in the NAT with the *p*-value (0.001) (Fig. 2B). Between the BC and NAT classes, an ROC curve was generated and the AUC score was shown. The marker of down-regulated miR-4510 indicated a significant AUC value and the AUC value of which amounted to 0.8823 (Fig. 2C). To find the prognostic correlation between the miR-4510 expression and patient overall survival (OS), Kaplan–Meier curve was designed and showed that a significantly prognostic association was found between the expression level of miR-4510 and patient overall survival, *p*-value (0.0003) (Fig. 2D).

### Analysis of SNPs in the mature sequence of miR-4510

**Figure 3:**
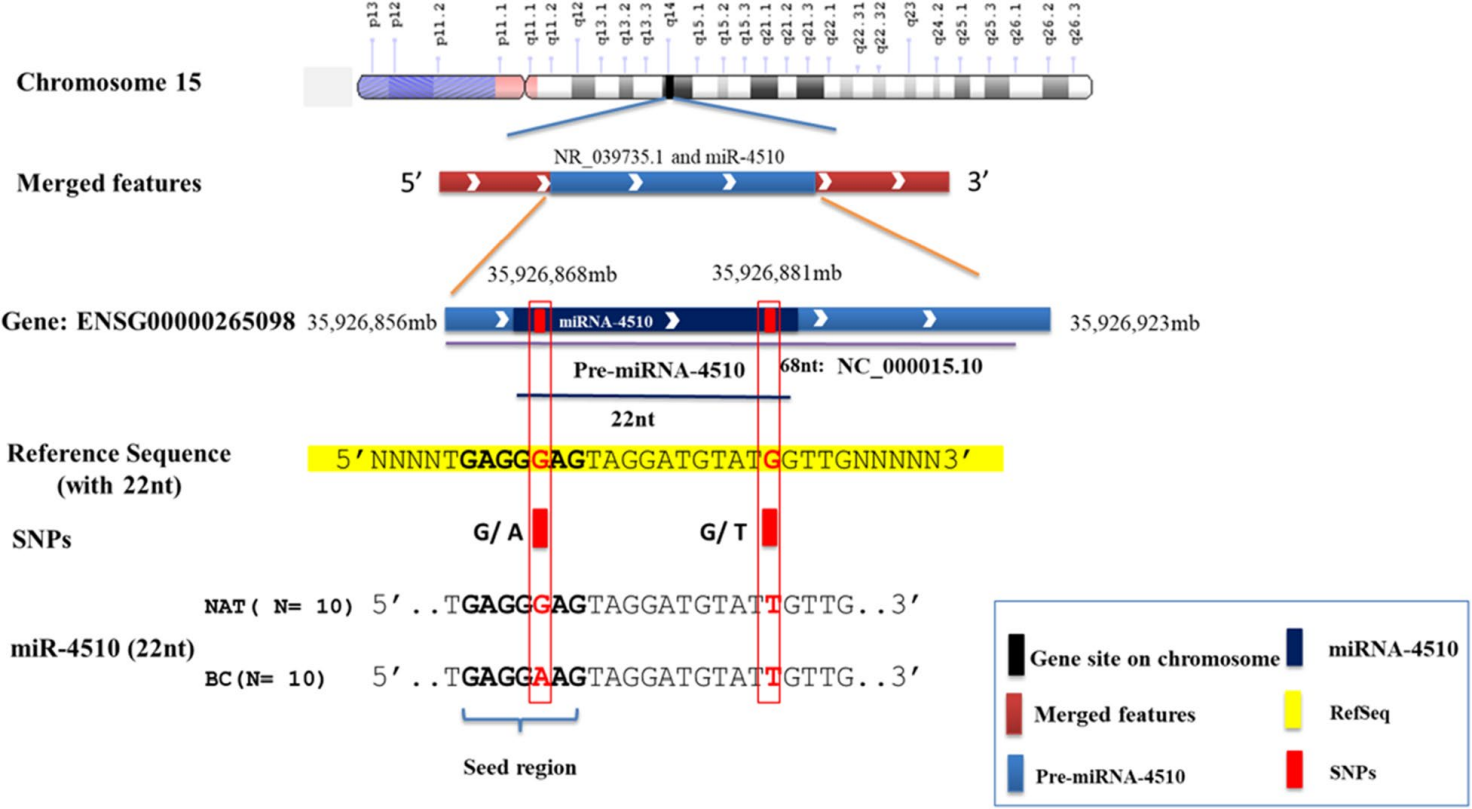
A schematic depiction of miR-4510 gene locus. Tracks represent the chromosomal regions, miR-4510 gene, SNP positions, and most predominant RefSeq. Comparison of mature sequences of miR-4510 in BC, and NAT with the Reference Sequence (RefSeq). Guanin (G) was changed into Thymine (T) in BC and NAT and Guanin (G) inside the seed sequence was also changed into Adenine (A) in BC compared to the RefSeq.

**Figure 4:**
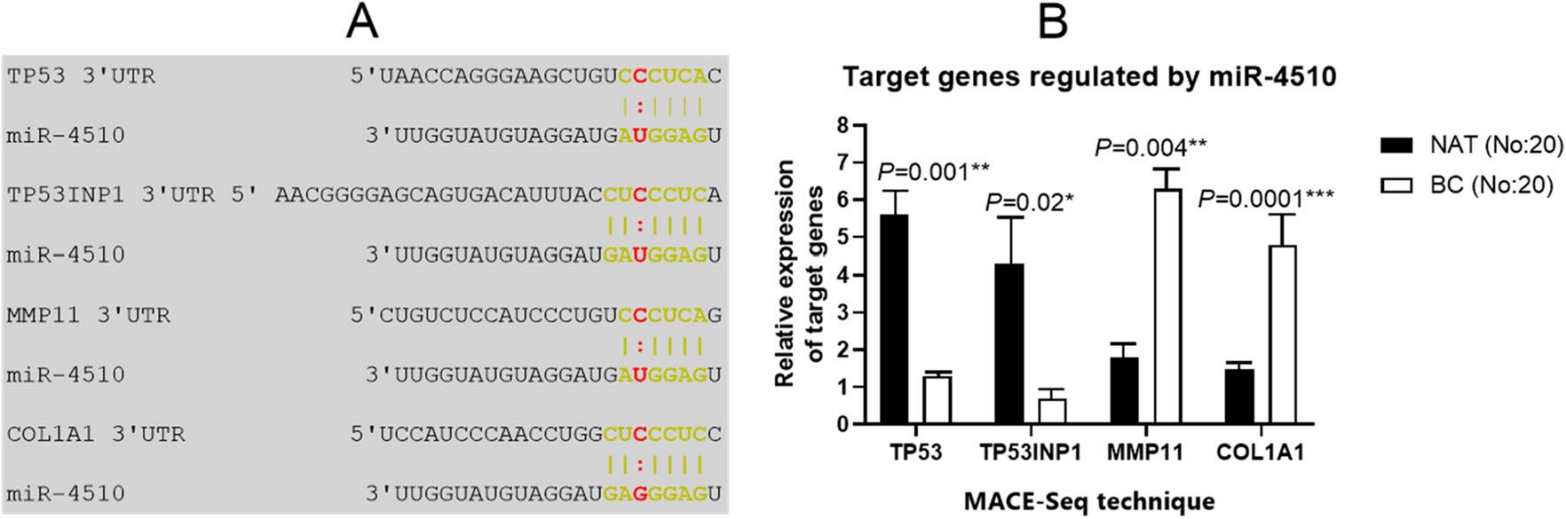
MRNA**-**miR-4510 interaction and differential expression of its target genes. (**A**) Complementarity of the 3′UTR of target sequence to the miR-4510 binding site which highlighted in green color. A polymorphism (A:C) was upper letter and highlighted in red color. (**B**) Differential expression level of miR-4510 target genes in BC participants compared to NAT. *P < 0.05, **P < 0.01.

The location of miR-4510 gene is located on the chromosome of number 15 and between 35,926,856 and 35,926,923 nts. The miR-4510 was sequenced and was 68nt in length. The mature sequence of which is 22nt (Fig. 3). About 23 single nucleotide polymorphisms (SNPs) have been recently identified in this (https://www.ncbi.nlm.nih.gov/nuccore/MZ387989). In this study, we sequenced the mature sequence of miR-4510 for 20paired samples of Kurdish patients to determine if a single nucleotide polymorphism (SNP) is present. The sequencing results showed that the mature sequence of which consisted of 22 nucleotides. Within the mature sequence, three novel polymorphisms were discovered; two within cancerous and one in non-cancerous samples. Two polymorphisms were occurred on a Kurdish population level, which Guanine (G) in BC and NAT cells was changed into Thymine (T) at the same location of 35,926,881, as compared against the reference sequence (RefSeq) in order to identify the polymorphisms in miR-4510. The Poly-miRTSs were detected outside the miR target position (Fig. 3). Another polymorphism, which is the change of Guanine (G) in BC into the Adenine (A) at the location of 35,926,868, was detected within the miR-4510 binding site to the mRNA 3′ UTR. This SNP may affect the interaction between miR-4510 and target mRNAs, including *TP53*, *TP53INP1*, *MMP11*, and *COL1A1* that are implicated in the various vital processes in the breast carcinogenesis, such as cell growth, development proliferation, differentiation, invasion, apoptosis, inflammation, and stress response (Fig. 4A). MicroRNA targeting is mostly achieved through specific base-pairing interactions between the 5′ end (seed region) of the miRNA and sites within coding and untranslated regions (UTRs) of mRNAs; target sites in the 3′ UTR diminish mRNA stability. The SNPs were submitted to dbSNP database, they will be released in dbSNP build (B156).

### mRNA-miR-4510 interaction and differential expression level of target genes

There is evidence that multiple genes can be regulated by a single miRNA. In the present study, *TP53*, *TP53INP1*, *MMP11*, and *COL1A1* were found to have the complementary base pairing to miR-4510 based on many target-predicted tools. it was also found that two polymorphisms were present in the miR-4510 of the BC samples. Because the change 35,926,868 (G/A) in the miR-4510 seed region in BC cells occurred, this cannot perfectly interact with the 3′-untranslated regions (UTR) of these mRNAs, thus increase the expression level of these genes and also impact on one or many cellular processes (Fig. 4A). The MACE-sequencing results showed that the gene of *MMP11* (*p*-value = 0.004) and COL1A1 (*p*-value = 0.0001) in BC tissues were significantly up-regulated as compared to the normal tissues and also the expression level of *TP53* (*p*-value = 0.001), and *TP53INP1* (*p*-value = 0.02) was significantly down-regulated (Fig. 4B). Table 5 also provided the information and experimental data on these genes. Eleven predicted target sites showed that these four genes had the binding sites to miR-4510 seed site.

**Table 5:** Target predicted genes had complementary bases with the miR-4510 seed site.

Targets	Gen ID	Description	No. of tools predicted the gene as has-mir-4510 target
*TP53*	ENST00000420246	Tumor protein p53	10
*TP53INP1*	ENST00000448464	Tumor protein p53 inducible nuclear protein 1	10
*MMP11*	ENST00000215743	Matrix metallopeptidase 11	10
*COL1A1*	ENST00000225964	Collagen type I alpha 1 chain	10

### Survival analysis

**Figure 5:**
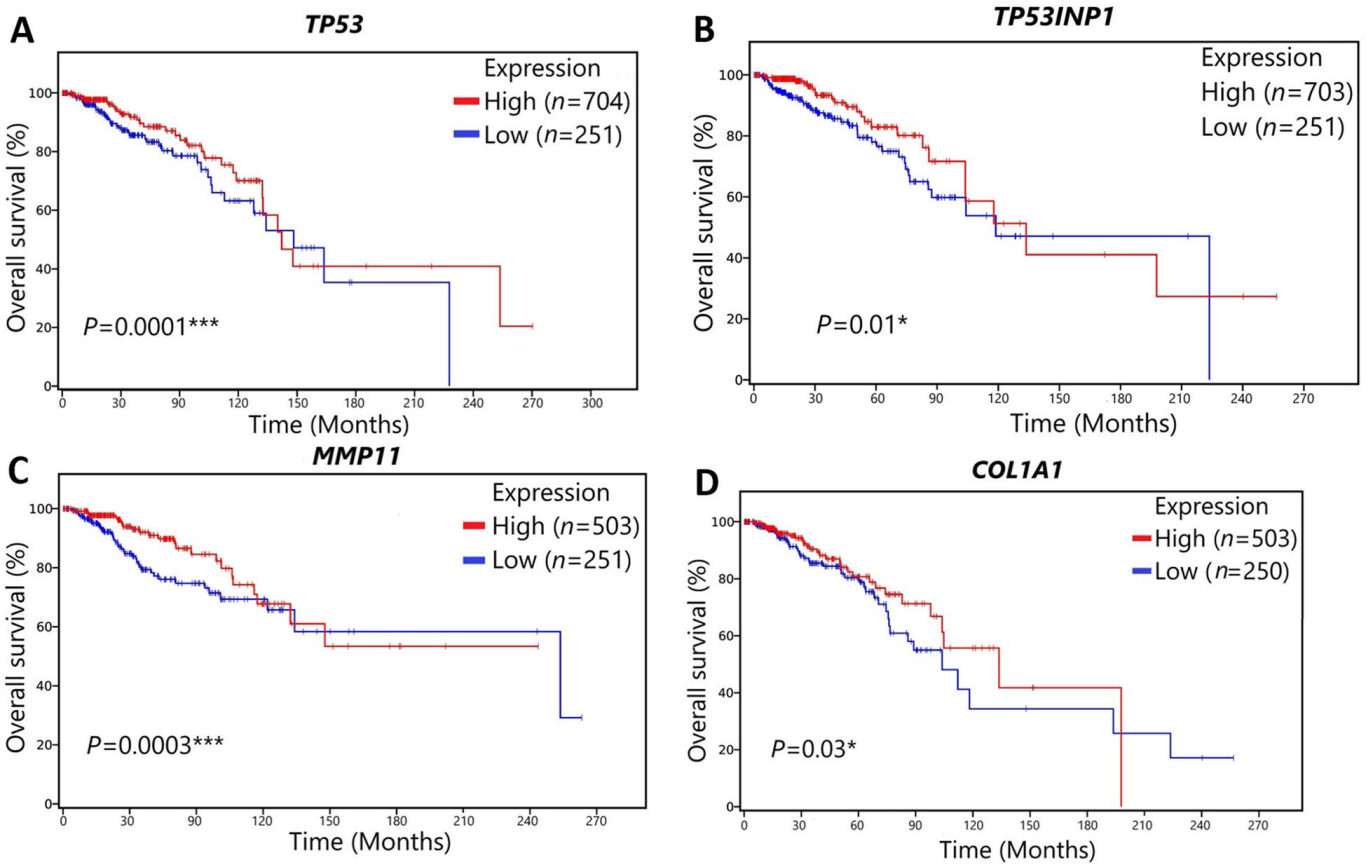
The significant effect of the expression value of target genes (*TP53*, (**A**)*, TP53INP1* (**B**), (**C**), *MMP11* and *COL1A1* (**D**)) on patient with BC by the Kaplan–Meier survival curves shows that patients with BC were split into 2 classes according to their expression values.

We designed Kaplan–Meier (KM) survival curve to visualize the survival relation between the expression value of miR-4510 target genes and BC patient survival with *p*-value < 0.05. Overall survival (OS) data was collected from the Cancer Genome Atlas (TCGA) database for more than 750 participants and then analyzed by the R program. The BC patients were split into two class, which are high and low expression. Figure 5 showed that differential expression of the gene of *TP53* (*p*-value = 0.0001) and *MMP11* (*p*-value = 0.0003) had a significant correlation with BC patient survival; whereas, the differential expression of *TP53INP1* (*p*-value = 0.01) and *COL1A1* (*p*-value = 0.03) *was* highly correlated with BC patient overall survival.

### Gene ontology (GO) enrichment analysis

**Figure 6:**
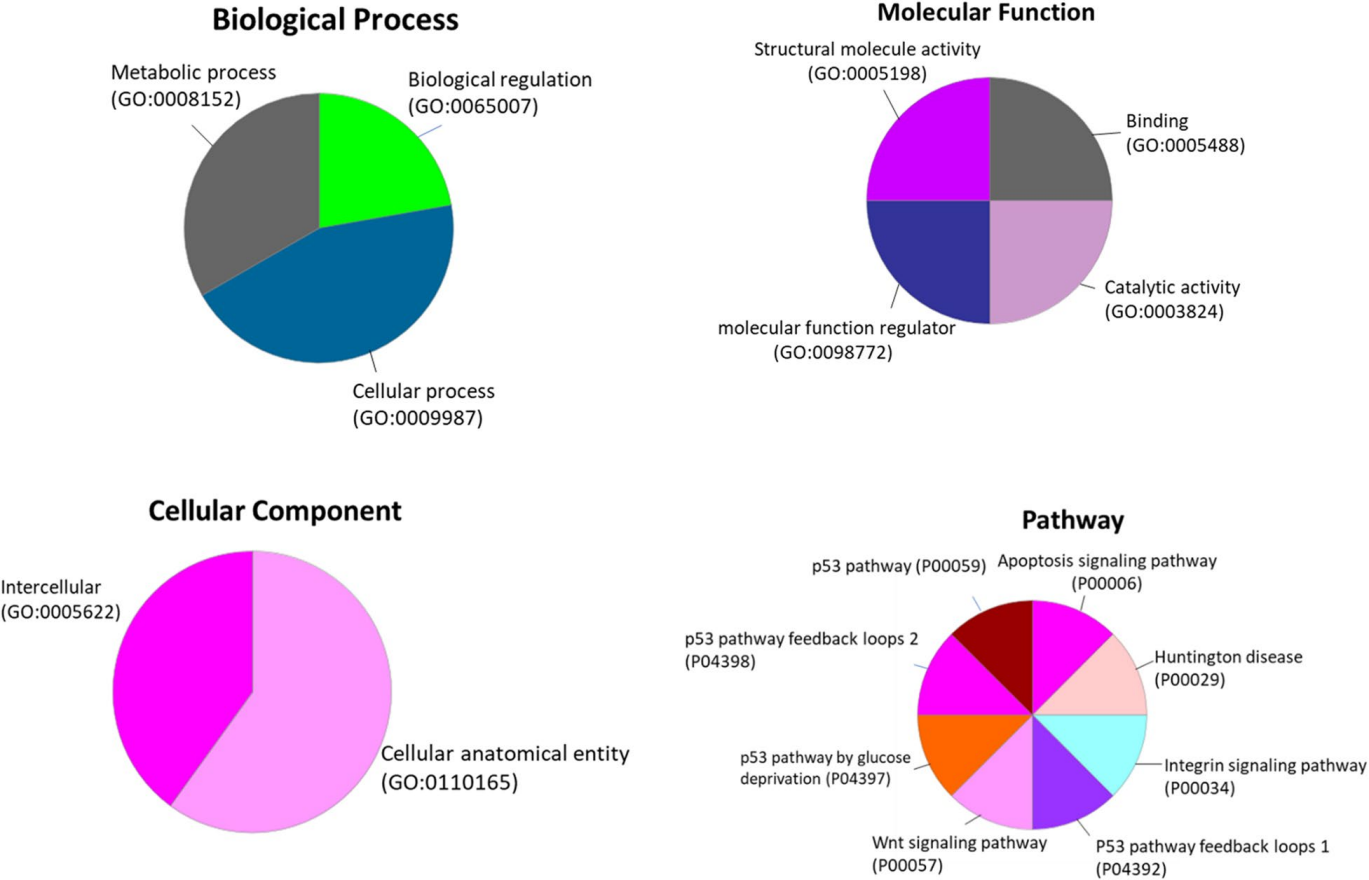
Gene ontology functional enrichment analyses of differentially expressed genes (DEGs) for targeted genes of hsa-miR-4510.

Based on the DAVID and GenXPro databases, GO enrichment analysis of differentially expressed genes (DEGs) was executed for determination of the biological role of the targets of hsa-miR-4510 in the BC. The enrichment analysis showed that up-regulation and down-regulation of *TP53, TP53INP1, MMP11,* and *COL1A1* played a key role in the biological processes of cells. The down-regulated DEG of *TP53*, *TP53INP1* and up-regulated DEG of *MMP11*, and *COL1A1* were enriched in the GO_terms, such as Biological Process (BP), Molecular Function (MF), Cellular component (CC), and Pathway (Fig. 6).

## Discussion

In the present study, MACE-sequencing-based protein coding RNAs and small RNA-Seq-based sncRNAs were analyzed to determine the differential expression profiles in BC paraffin tissue compared with NAT. In BC tissue, miR-4510 was recognized as a novel tumor suppressor and was also found to have a lower expression than in NAT. In addition, 3 poly-miRTSs (2G > T, 1G > A) were newly identified in the mature sequence of miR-4510. Two of them were found in both BC and NAT in the same site of the miR-4510 seed sequence, and these were occurred on the Kurdish population level; whereas, another occurred in the mature sequence of miR-4510 in BC. Moreover, four genes; including *TP53*, *TP53INP1*, *MMP11*, and *COL1A1*, were found as novel putative targets of miR-4510 and also negatively over-expressed. As a result, miR-4510 was hypothesized through the overexpression of these genes in BC cells to function as a tumor suppressor. Cell growth, proliferation, migration and invasion of BC cells were blocked by miR-4510 up-regulation. The results of this study showed that miR-4510 could be examined as a new BC biomarker.

Recently, small RNAs have been paid more attention by researchers. Especially miRNAs have received the most attention because dysregulation of the miRNAs has been found to affect cellular processes at various levels of gene expression. In various normal and malignant cells, they can regulate one or more protein-coding and/or none-coding genes^33,34^. Recently, many molecular biological techniques, such as NGS sequencing, DNA microarrays, MACE-sequencing, small RNA-Seq, qRT-PCR, and RNA-sequencing, have been developed to identify differential expression of the total RNA molecules in human diseases, particularly human cancers^35–39^. One of the RNAs that function as a gene regulator is the miRNA. Recently, many studies have documented those multiple miRNAs, such as miR-146a/b, miR-100, miR-200c, miR-107, miR-124, miR-122, miR-205-3p, and miR-99a-5p, are continuously down-expressed in the breast malignant cells and act as tumor-suppressor^34,37,40–44^. In the present study, several miRNAs in BC tissues were found to be down-regulated compared to NAT, but only we focused on miR-4510 and their putative target genes. Recent studies have found that miR-4510 is down-expressed in colorectal, recurrent breast and bladder carcinoma^45–47^. Another recent study has documented that Glypican-3 (*GPC3*) is targeted by miR-4510 in liver malignancies; including hepatocellular carcinoma (HCC) and hepatoblastoma. The *GPC3* is found to be negatively expressed^48^. A new study have also shown that miRNA-4510 inhibits development of liver cancer by targeting *RAF1* and *RAS*/*RAF*/*MEK*/*ERK* signaling malfunction^49^.

However, the biological function of each *TP53*, *TP53INP1*, *MMP11*, and *COL1A1*was determined according to the GO terms enrichment with DEGs. These were enriched in the GO terms of the extracellular matrix disassembly, negative regulation of apoptotic process, and protein binding. Two recent studies revealed that the overexpression of *IGF1R* and CD44 could decrease the apoptosis processes of the BC cells and also increased the growth and invasion of BC cells^50,51^. The miR-4510-regulated putative targets and their role in the breast malignancy were aimed to newly elucidated in this study. Based on the eleven prediction programs of miRNA target prediction, four novel genes (*TP53*, *TP53INP1*, *MMP11*, and *COL1A1*) were selected to be targeted by miR-4510. Differential expression of these the BC cells were found to be up-regulated in the MACE-sequencing results. They were also found to be closely correlated with favorable or poor prognosis. *TP53* gene was found to be important for regulation of apoptotic process in breast normal cells; whereas, up to 50% of breast tumors down expressed this gene, which decreased the cell apoptotic process, Farabaugh and his colleagues reported^52^. A study showed that *TP53* was regulated by miR-145. The activation of TP53 can then induce the expression of miR-145, forming a death-promoting loop between miR-145 and TP53^53^. A new study has found that this gene is also regulated through hsa-miR-1269a in BC cells under hypoxia^54^. However, in human BC, MicroRNA-155-5p promotes tumor development and contributes to paclitaxel resistance via TP53INP1. Another new study reports that NR 027471 is a lncRNA that acts as a ceRNA for miRNA-8055. Regulating the expression of TP53INP1 leads to the suppression of osteosarcoma^55^. Another new study revealed that miR-125b in BC acts as a tumor suppressor by targeting MMP11^56^. Up-regulation of this was found to promote the cell proliferation, migration and invasion, Geng and his colleague has now shown^57^. A new study has also found that MMP11 could be delivered to BC cells via exosomes. MMP11 also acted as a sponge for miR-153-3p, regulating ANLN expression and boosting lapatinib resistance in BC cells, resulting in therapeutic targets for BC treatment^58^. However, it was found that miR-196b-5p down-regulation inhibits BC cell proliferation, migration, invasion and metastasis through modulating COL1A1^59^. By targeting COL1A1, down-regulation of the let-7i promotes gastric cancer invasion and metastasis^60^. Bioinformatics study of COL1A1 as a potential therapeutic target for gastric cancer, which is regulated by miR-129-5p^61^.

However, regulatory SNPs (rSNPs) in the short non-coding RNAs have been recently paid more attention, especially those within mature sequence and/or seed sequence of miRNAs have the most attention in human cancers. Moszynska and his colleagues reviewed^35^. A study found that poly-miRTSs within the binding sites can generate a new mRNA target region or impair the existing target region^62–64^ . For example, Mdm2 is a significant oncoprotein which negatively modulates the p53 protein. Up-regulation of this protein is found to result in cancer growth^65^. Two recent experiments showed that the change in the *MDM4* 3′-UTR may contribute to a low risk of different cancers. The existence of the C minor allele (SNP rs4245739 A.C) in the *MDM4* 3′-UTR can reduce the cancer risk, and the metastasis progression. The introduction of this C minor SNP has been also shown to generate a novel binding region for miR-191 and miR-887-3p and thus this causes a low level of *MDM4* protein^66,67^. Some studied have shown that the poly-miRTSs can affect post-transcriptional gene expression and transcriptional regulation. they can result in changes in cellular processes at various levels of gene regulation. Approximately 29 poly-miRTSs within miR-4510 gene has been recorded in National Center of Biotechnology Information (NCBI) and no fully researchers have found the influences of these SNPs. poly-miRTSs fall within a miR binding region has a key effect on miRNA-regulated genes through strengthening or weakening the mRNA: microRNA interaction^68^. A new study has documented that a SNP located within the *COPD* target region of miR-4510 and led to the new functional role in lung disease^69^. another study has reported that *CDH7*, *P53*, and *MRE11A* genes targeted by mi4510 in colon cancer. expression of these was found to be over when miR-4510 binding site was altered^70^.

## Conclusion

To sum up, this research has shown that the level of miR-4510 expression in BC was lower compared to NAT, and that down-expression of miR-4510 could increase cellular processes such as cell development, proliferation, and invasiveness of BC cells by inducing *TP53*, *TP53INP1*, *MMP11*, and *COL1A1*genes. It was identified that Poly-miRTSs within the seed sequence of miR-4510 can impair the existing target site and thus lead to the protein instability of these genes. The current study has also shown that BC cells had a negative increase in the level of expression of these genes. Overexpression of these gene can affect the BC development. Finally, this topic needs further studies in future researches because the regulatory role and Poly-miRTSs of miR-4510 in BC can be complicated,

## Data Availability

The data of MACE-sequencing available at http://www.ncbi.nlm.nih.gov/bioproject/900415. While sequencing data available at https://www.ncbi.nlm.nih.gov/nuccore/MZ387990.

## Abbreviations

MiRNA/miR: MicroRNA
FFPE: Formalin-Fixed Paraffin-Embedded
BC: Breast Cancer
NAT: Normal adjacent tissue
ncRNA: Non-coding RNA
sRNA: Small RNA
MACE: Massive Analysis of cDNA Ends
rSNP: Regulatory single nucleotide polymorphism
ER: Estrogen Receptor
PR.: Progesterone Receptor
HER2: Human Epidermal Growth Factor Receptor 2
**–**: Negative
** + **: Positive
OS: Overall Survival
TCGA: The Cancer Genome Atlas
FDR: False Discovering Rate
FC: Fold Change
DEncRNAs: Differentially expressed ncRNAs
GO: Gene Ontology
CC: Cellular Compartment
BP: Biological Process
MF: Molecular Function
